# hucMSCs treatment prevents pulmonary fibrosis by reducing circANKRD42-YAP1-mediated mechanical stiffness

**DOI:** 10.18632/aging.204805

**Published:** 2023-06-16

**Authors:** Haitong Zhang, Qi Zhu, Yunxia Ji, Meirong Wang, Qian Zhang, Weili Liu, Ruiqiong Li, Jinjin Zhang, Pan Xu, Xiaodong Song, Changjun Lv

**Affiliations:** 1 Department of Respiratory and Critical Care Medicine, Binzhou Medical University Hospital, Binzhou Medical University, Binzhou 256603, China; 2 Department of Cellular and Genetic Medicine, School of Pharmaceutical Sciences, Binzhou Medical University, Yantai 264003, China; 3 Department of Pathology, Binzhou Medical University Hospital, Binzhou Medical University, Binzhou 256603, China; 4 Department of Clinical Nursing, Binzhou Medical University Hospital, Binzhou Medical University, Binzhou 256603, China

**Keywords:** pulmonary fibrosis, hucMSCs, circRNA, mechanical stiffness, YAP1

## Abstract

Idiopathic pulmonary fibrosis (IPF) is a fibrosing interstitial pneumonia of unknown cause. The most typical characteristic of IPF is gradual weakening of pulmonary elasticity and increase in hardness/rigidity with aging. This study aims to identify a novel treatment approach for IPF and explore mechanism of mechanical stiffness underlying human umbilical cord mesenchymal stem cells (hucMSCs) therapy. Target ability of hucMSCs was examined by labeling with cell membrane dye Dil. Anti-pulmonary fibrosis effect of hucMSCs therapy by reducing mechanical stiffness was evaluated by lung function analysis and MicroCT imaging system and atomic force microscope *in vivo* and *in vitro*. Results showed that stiff environment of fibrogenesis caused cells to establish a mechanical connection between cytoplasm and nucleus, initiating expression of related mechanical genes such as Myo1c and F-actin. HucMSCs treatment blocked force transmission and reduced mechanical force. For further exploration of mechanism, ATGGAG was mutated to CTTGCG (the binding site of miR-136-5p) in the full-length sequence of circANKRD42. Wildtype and mutant plasmids of circANKRD42 were packaged into adenovirus vectors and sprayed into lungs of mice. Mechanistic dissection revealed that hucMSCs treatment repressed circANKRD42 reverse splicing biogenesis by inhibiting hnRNP L, which in turn promoted miR-136-5p binds to 3′-Untranslated Region (3′-UTR) of YAP1 mRNA directly, thus inhibiting translation of YAP1 and reducing YAP1 protein entering nucleus. The condition repressed expression of related mechanical genes to block force transmission and reduce mechanical forces. The mechanosensing mechanism mediated directly by circANKRD42-YAP1 axis in hucMSCs treatment, which has potential general applicability in IPF treatment.

## INTRODUCTION

Idiopathic pulmonary fibrosis (IPF) is a chronic, fibrosing interstitial pneumonia of unknown cause [1]. Several potential risk factors, such as aging and genetic susceptibility; chronic administration of some drugs, such as cyclophosphamide, bleomycin, and procainamide; and environmental exposure, including air pollution and fungal and bacterial infections [2], can act on various types of lung cells and enhance the risk of developing IPF. Of these risk factors, aging is considered an independent risk factor for IPF [3]. The most typical characteristic of IPF is the gradual weakening of pulmonary elasticity and the gradual increase in hardness/rigidity with the increasing of ages, leading to the patient dying of respiratory dysfunction. Increased in pulmonary hardness can be caused by many mechanical factors, such as elevated mechanical tension, stiffness, stretch, and cellular adhesion and density [4, 5]. These mechanical factors of lung tissues have a great impact on the biological behavior of lung cells, such as fibroblast–myofibroblast differentiation, myofibroblast proliferation, and migration. Therefore, understanding the mechanism and searching for a treatment targeting the hardness of lung tissues are necessary and exigent.

Human stem cells provide unprecedented opportunities for cell therapies against intractable diseases and injuries. Some stem cell therapies, such as embryonic stem cells and induced pluripotent stem cells, are already being used in clinical trials [6]. Human umbilical cord mesenchymal stem cells (hucMSCs) are a good source for stem cell therapy and used to treat many diseases, including diabetes, spinal cord injury, and chronic myeloid leukemia [7–9]. Wang et al. [10] constructed a stable overexpressing interleukin 10 hucMSCs to alleviate high-fat diet-induced obesity. Gao et al. [11] combined hucMSCs with cell sheet technology to treat myocardial infarction. In pulmonary fibrosis, hucMSCs exhibit immunoregulation to attenuate pulmonary fibrosis by interacting with macrophages [12]. hucMSCs differentiate into type 2 alveolar epithelial cells to protect mouse pulmonary fibrosis through β-catenin-regulated cell apoptosis [13]. Exosomes derived from hucMSCs are a potential therapy of silica-induced pulmonary fibrosis by targeting TGFBR1 [14]. Circular RNAs (circRNAs) are covalently closed, endogenous RNAs with no 5′ end caps or 3′ poly(A) tails and are involved in different biological and pathophysiological processes, including pulmonary fibrosis [15, 16]. However, the hardness mechanism underlying circRNA-mediated hucMSCs therapy for pulmonary fibrosis has never been reported.

circANKRD42 is a profibrotic factor and enhances the mechanical stiffness in IPF depending on miR-136-5p and YAP1. It also is a promising biomarker and a potential therapeutic target related to the mechanical stiffness for IPF treatment [5]. Whether the circANKRD42-mediated mechanical stiffness signal pathway is the target of hucMSCs treatment is unclear. In this study, cell/tissue stiffness and cytoskeleton tension were taken as the examples of mechanical stiffness and the formation of pulmonary fibrosis was taken as a pointcut of IPF. The stiffness mechanism underlying circANKRD42-mediated hucMSCs therapy in pulmonary fibrosis was thoroughly investigated. It provides a potential treatment for pulmonary fibrosis and further shows that circANKRD42 is a promising biomarker. This study provides a novel therapeutic target and depicts the mechanical stiffness mechanism underlying circRNA-mediated hucMSCs therapy for pulmonary fibrosis.

## RESULTS

### Identification of hucMSCs

**Figure 1:**
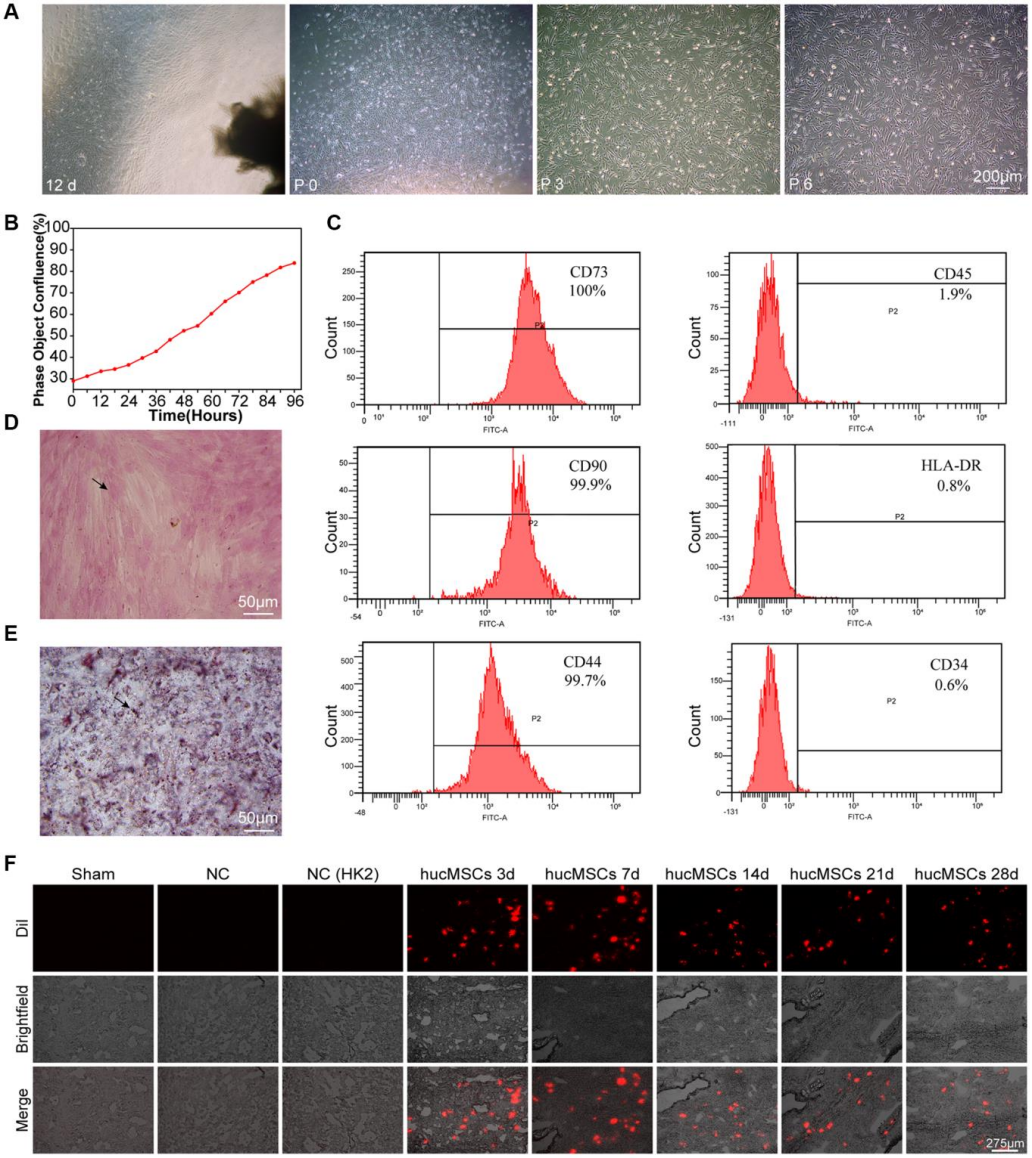
**Culture and identification of hucMSCs.** (**A**) Morphological observation of hucMSCs at different times. After 12 days of primary cultivation, cells developed around the tissue block. Zero generation of hucMSCs. Third generation of hucMSCs. Sixth generation of hucMSCs. (**B**) Growth curve of the third generation of hucMSCs showing their excellent growth as recorded by an IncuCyte S3 instrument. (**C**) Flow cytometry indicated that the positive rate was 100% for CD73, 99.90% for CD90, and 99.70% for CD44. The positive rate was 0.6% for CD34, 1.9% for CD45, and 0.8% for HLA-DR. (**D**) After 14 days of osteogenic induction, a positive reaction was noted between calcium nodus alizarin red stain and the osteogenic inducer. (**E**) After 28 days of adipogenic induction, fat droplets of verified size were positive for oil red O staining. (**F**) Images of cell membrane fluorescent probe revealing that hucMSCs were retained in the lungs. Mice injected with serum-free medium were used as sham. Mice injected with hucMSCs without fluorescent labeling were used as NC-1 to prove the absence of biological autofluorescence. Mice injected with human kidney 2 (HK-2) cells with labeled fluorescence were used as NC-2 to prove the absence of fluorescence dye leakage.

Light microscopy, IncuCyte S3 instrument detection, flow cytometry, and osteogenesis and adipocyte induction were performed to identify whether the isolated cells are hucMSCs. After 12 days of primary cultivation, the scattered transparent cells developed around the tissue block. The cells gradually became spindle and fused into pieces. The tissue block was removed, and the cells were called generation P_0_. The transparent dots gradually became long, and their number gradually increased. On the 9th day after the tissue block adhered to the wall, spindle cells gathered around the tissue block. On the 12th day, the cells around the tissue block fused into pieces (Figure 1A). The real-time growth of the third generation of cells was monitored using an IncuCyte S3 instrument. The growth curve showed that the cells grew well (Figure 1B). Flow cytometry was performed to measure the level of surface markers on the cells. The result showed that the cells expressed CD73, CD90, and CD44 but not CD34, CD45, and human leukocyte antigen DR HLA-DR, (Figure 1C). Osteogenic differentiation, which indicates the formation of bone matrix, was detected by alizarin red staining (Figure 1D). Adipogenesis differentiation was determined by Oil Red O staining, and numerous intracellular lipid droplets were observed (Figure 1E). Finally, hucMSCs were labeled with a cell membrane dye Dil, and then the labeled cells were injected into the mice via the tail vein. The fluorescence signals of lung section were captured after 3, 7, 14, 21, 28 days of treatment. The results showed that remarkable fluorescence signals were recorded in the lung of mice, indicating the strong targeting ability of hucMSCs to lung. Although the number of surviving stem cells decreased at a certain period after hucMSCs injection into the mice, some viable hucMSCs were still present on the 28th day after hucMSCs treatment (Figure 1F). The above data elucidated that hucMSCs were successfully cultured and they can be retained in the lungs.

### hucMSCs treatment alleviated pulmonary fibrosis by reducing YAP1-mediated mechanical stiffness

**Figure 2:**
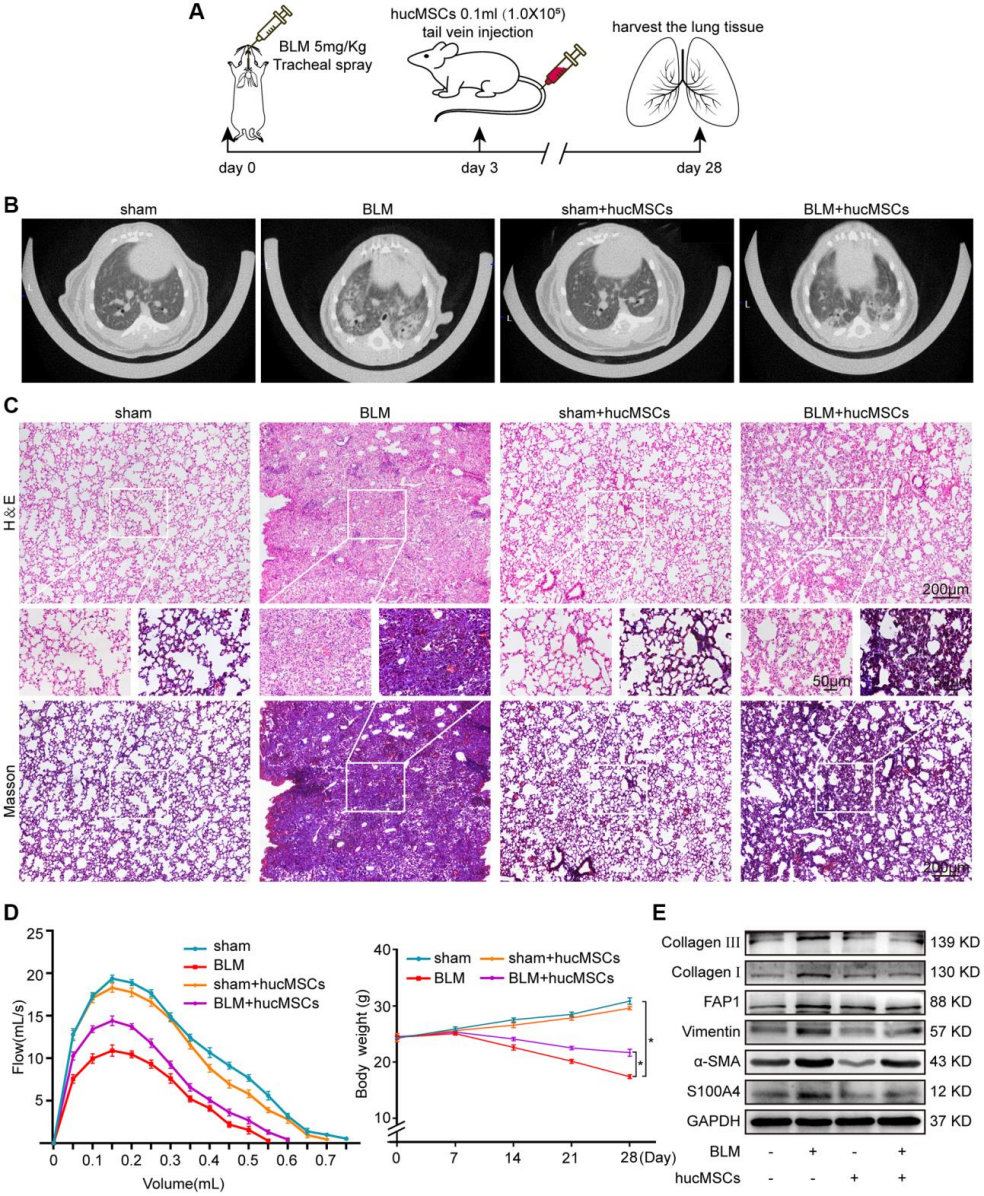
**hucMSCs treatment alleviated pulmonary fibrosis in mice.** (**A**) Schematic of the administration of BLM or hucMSCs into mice. (**B**) MicroCT images for small animal depicting that the BLM group had honeycomb-like changes and uneven patchy shadows compared with the sham group. These fibrotic symptoms were remarkably alleviated in the hucMSCs-treated group. (**C**) H&E and Masson staining results indicated that excess collagen was deposited, alveolar structure was damaged, and the alveolar wall was thickened in the BLM group. Meanwhile, collagen deposition was reduced and the alveolar structure was clear and complete in the hucMSCs-treated group. (**D**) FVC result showed that hucMSCs treatment improved the lung function of mice compared with the sham group. Body weight monitoring revealed that hucMSCs treatment suppressed the body mass loss compared with that in the sham mice. (**E**) Western blot analysis indicated that hucMSCs inhibited the expression of fibrotic proteins such as α-SMA, vimentin, collagen I, and collagen III and the differentiation-related proteins S100A4 and FAP1. Each bar represents the mean ± SD; *n* = 6; ^*^p < 0.05.

The antipulmonary fibrotic effect of hucMSCs treatment was assessed *in vivo* and *in vitro*. hucMSCs were injected into the mice through the tail vein after 3 days of bleomycin (BLM) spraying (Figure 2A). MicroCT images showed that the lungs of BLM-treated mice had evident honeycomb lung and uneven patchy shadows. These fibrotic symptoms were dramatically attenuated under hucMSCs treatment (Figure 2B). H&E and Masson staining results revealed that compared with those in BLM group, the collagen deposition was reduced and the alveolar structure was clear and complete in hucMSCs-treated group (Figure 2C). Lung function testing showed that the forced vital capacity (FVC) of hucMSCs-treated mice was improved. Body weight monitoring reflected that hucMSCs treatment suppressed the body mass loss compared with that in the sham mice (Figure 2D). Western blot analysis clarified that hucMSCs repressed the expression of fibrotic proteins such as α-SMA, vimentin, collagen I, and collagen III. Differentiation-related proteins, including S100 calcium binding protein A4 (S100A4) and fibroblast activation protein alpha (FAP1), were also inhibited by hucMSCs (Figure 2E). The above data indicated that hucMSCs treatment alleviated pulmonary fibrosis *in vivo*.

**Figure 3:**
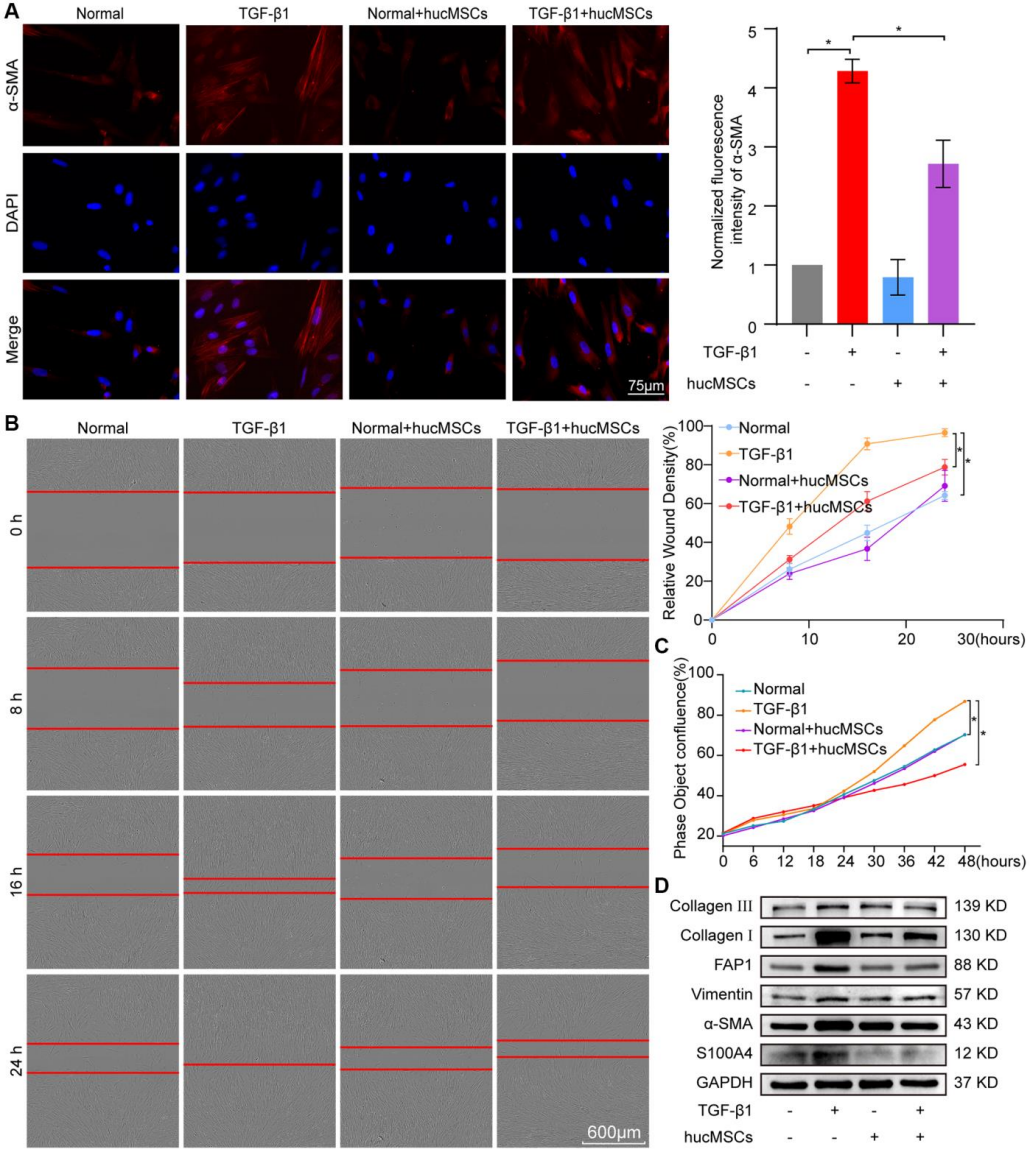
**hucMSCs treatment repressed pulmonary fibrosis in TGF-β1-treated MRC-5 cells.** (**A**) Immunofluorescence staining showed the spindle-shaped cells, enhanced cell proliferation, and a-SMA increase in the TGF-β1 group. hucMSCs treatment repressed a-SMA and cell proliferation. Blue indicates nucleus marked with DAPI, and red indicates a-SMA in cytoplasm. (**B**) Cell scratch assay monitored with an IncuCyte S3 instrument validated that the migration was promoted by TGF-β1 treatment and inhibited by hucMSCs treatment. (**C**) IncuCyte S3 instrument detected showed that TGF-β1 promoted cell proliferation compared with that in the normal group. hucMSCs treatment weakened cell proliferation compared with that in the TGF-β1-treated group. (**D**) Western blot analysis revealed that the expression of FAP1, S100A4, α-SMA, vimentin, collagen I, and III substantially decreased in the hucMSCs-treated group compared with that in the TGF-β1-treated group. Each bar represents the mean ± SD; *n* = 6; ^*^p < 0.05.

The transforming growth factor-beta 1 (TGF-β1) activates human embryonic lung fibroblast (MRC-5) to differentiate into myofibroblast. Hence, this system was used as a cell model to further explore the role and mechanism of hucMSCs treatment against pulmonary fibrosis. Immunofluorescence staining showed that the cells became spindle shaped and revealed an increase in a-SMA in the TGF-β1 group. hucMSCs treatment repressed a-SMA expression (Figure 3A). Cell proliferation and migration were monitored using an IncuCyte S3 instrument, and the result showed that both processes were repressed by hucMSCs treatment compared with those in TGF-β1 treatment (Figure 3B, 3C). Western blot analysis elucidated that the levels of FAP1, S100A4, α-SMA, vimentin, and collagen I and III were decreased by hucMSCs treatment (Figure 3D). The above data indicated that hucMSCs treatment blocked pulmonary fibrogenesis *in vitro*.

**Figure 4:**
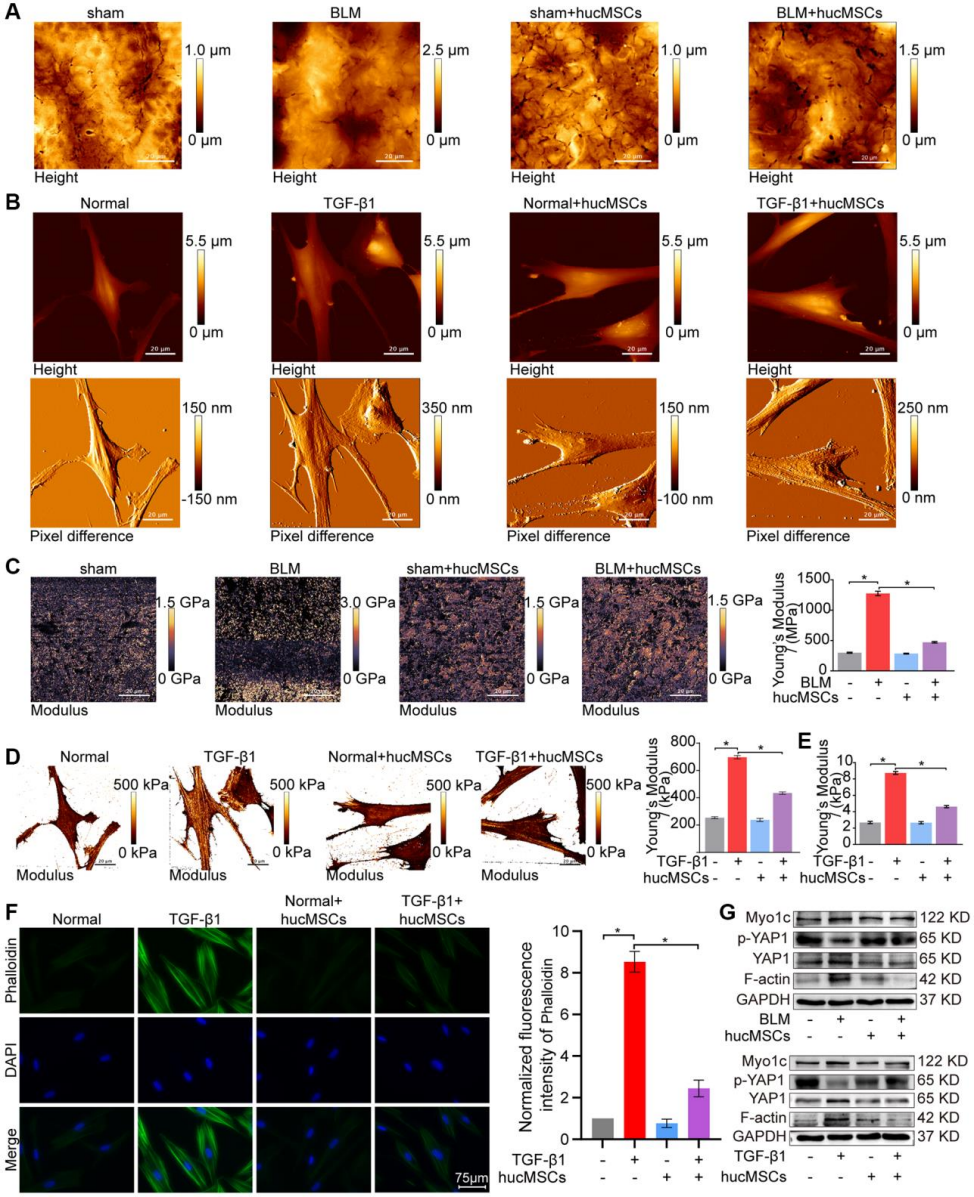
**hucMSCs treatment weakened mechanical stiffness in pulmonary fibrosis.** (**A**) AFM images showed that the surface of lung tissues was smooth in the sham group. BLM caused the lung surface to become rough and increased the lung tissue thickness. hucMSCs treatment improved lung tissue morphology. (**B**) AFM images displayed that the cells were spindle shaped with smooth surface in the normal group. After TGF-β1 stimulation, the cells became elongated and flat and their cell height/roughness increased. hucMSCs treatment improved the cell state. Stress fibers arranged in parallel along the main axis of cells were found in the TGF-β1 group. After hucMSCs intervention, these fibers were reduced. (**C**) Young’s modulus value increased in the BLM group compared with that in the sham group, and hucMSCs treatment decreased the Young’s modulus. The average Young’s modulus was 299.017 ± 21.021 MPa in the sham group, 1275.667 ± 89.502 MPa in the BLM group, 284.667 ± 18.778 MPa in the sham+hucMSCs group, and 471.183 ± 29.462 MPa in the BLM+hucMSCs group. (**D**) Young’s modulus increased in the TGF-β1 group compared with that in the normal group, and hucMSCs treatment decreased the Young’s modulus. The average Young's modulus was 254.583 KPa in the normal group, 697.217 KPa in the TGF-β1 group, 238.150 KPa in the normal+hucMSCs group, and 434.150 KPa in the TGF-β1+hucMSCs group. (**E**) Tests using colloid probe with ball stuck pressed cells were conducted to determine the reaction forces of cells based on Young’s modulus. The average Young’s modulus was 2.686 ± 0.349 KPa in the normal group, 8.742 ± 0.422 KPa in the TGF-β1 group, 2.660 ± 0.333 KPa in the normal+hucMSCs group, and 4.623 ± 0.349 KPa in the TGF-β1+hucMSCs group. (**F**) Cytoskeleton staining with FITC-phalloidin depicted that the cytoskeleton tension was aggravated by TGF-β1 treatment and alleviated by hucMSCs treatment. (**G**) hucMSCs treatment decreased YAP1, Myo1c, and F-actin expression and increased p-YAP1 expression in BLM-induced mice model and TGF-β1-activated cell model. Each bar represents the mean ± SD; *n* = 6; ^*^p < 0.05.

Finally, the effect of hucMSCs on cell/tissue stiffness and cytoskeleton tension was analyzed by atomic force microscopy (AFM), cytoskeleton staining, and Western blot. AFM was employed to examine the cell/tissue stiffness in response to hucMSCs treatment. AFM images exhibited that the lung tissues in the sham group had a smooth surface. BLM caused the lung surface to become rough and increase the lung tissue thickness. Meanwhile, hucMSCs treatment improved the lung tissue morphology (Figure 4A). AFM cell images displayed that the cells were spindle shaped with smooth surface in the normal group. After TGF-β1 stimulation, the cells became elongated and flat and the cell height/crudeness increased. hucMSCs treatment improved the cell state. Stress fibers arranged in parallel along the main axis of cells were found in the TGF-β1 group. After hucMSCs intervention, these fibers were reduced (Figure 4B). Young’s modulus value, also known as tensile modulus, was calculated from the displacements of boundary tissues and the force distribution expressing the changes of mechanical stiffness obtained using the AFM. Young’s modulus values revealed that the force measurement increased in the BLM/TGF-β1 group compared with that in the sham/normal group. In addition, hucMSCs treatment decreased the Young’s modulus (Figure 4C, 4D). Tests using colloid probe with ball stuck pressed cells were conducted to determine the reaction forces of cells. The reaction forces increased in the TGF-β1 group, and the reducing effect of hucMSCs treatment on the reaction forces was better than the TGF-β1 action (Figure 4E). Cytoskeleton tension was observed by FITC-phalloidin staining under a fluorescence microscope. The images indicated that the cytoskeleton tension was increased by TGF-β1 and decreased by hucMSCs treatment (Figure 4F). YAP is a sensor of the structural and mechanical features of the cell microenvironment, such as cellular stiffness, stretching, density, or confined adhesiveness [17, 18]. Force applied to the nucleus directly drives YAP nucleocytoplasmic shuttling via phosphorylation or dephosphorylation [19]. Our data showed that hucMSCs decreased YAP1 expression and increased p-YAP1 expression, suggesting that this treatment reduced the mechanical forces by blocking YAP1 nuclear entry. Myosin 1c (Myo1c) can sense cellular mechanical properties and function as a tension-sensitive anchor or transporter [20]. As an important component of cytoskeleton, F-actin is involved in cytoskeleton tension. The results showed that the expression of Myo1c and F-actin increased in pulmonary fibrosis *in vivo* and *in vitro* but was reduced by hucMSCs treatment (Figure 4G). The above data indicated that the stiff environment of fibrogenesis caused the cells to establish a mechanical connection between the cytoplasm and nucleus, initiating the expression of related mechanical genes such as Myo1c and F-actin. hucMSCs treatment blocked the force transmission and reduced the mechanical force by blocking YAP1 nuclear entry.

### hucMSCs treatment alleviated pulmonary fibrosis by regulating circANKRD42-miR-136-5p as the upstream of YAP1

**Figure 5:**
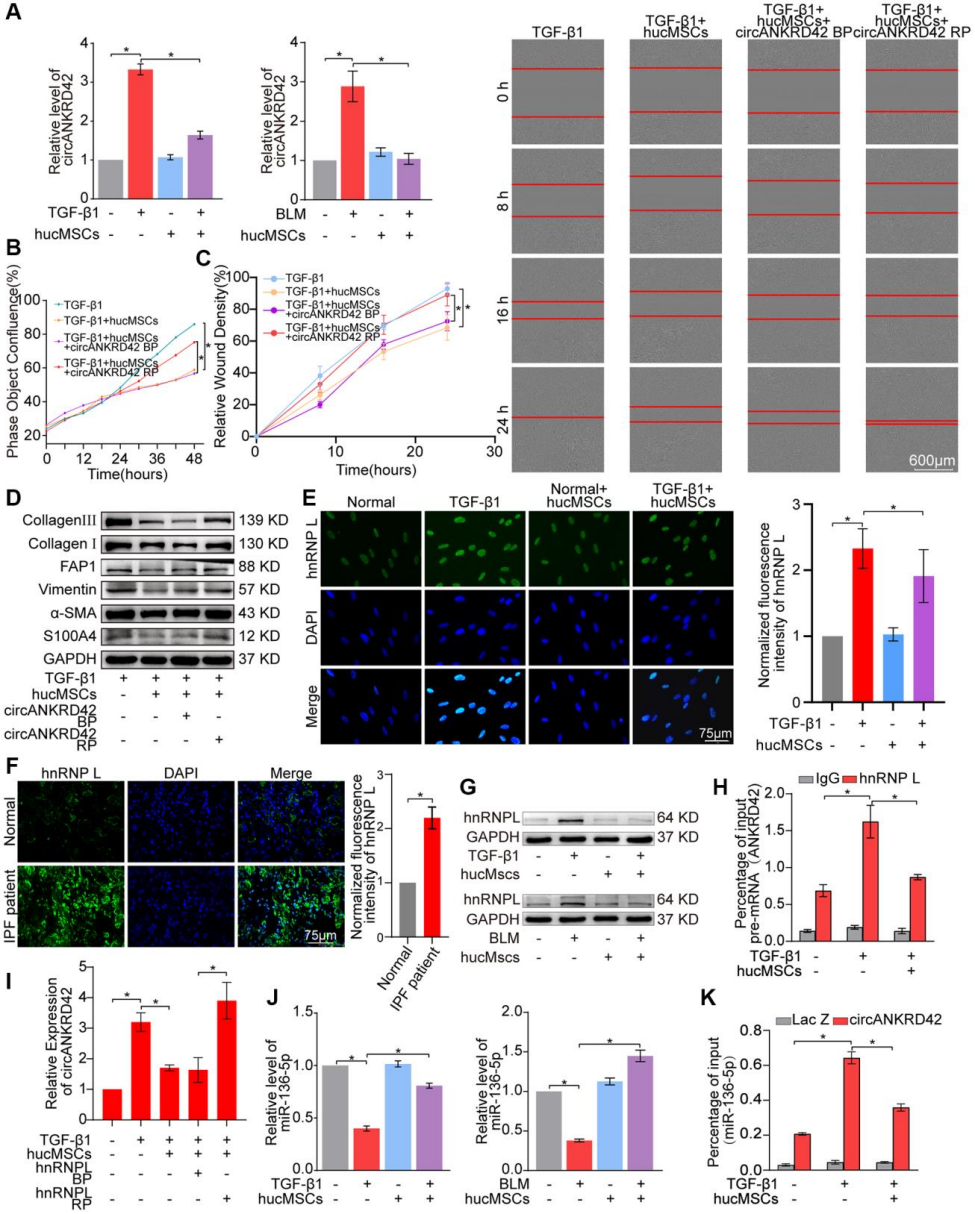
**hucMSCs treatment alleviated pulmonary fibrosis by preventing circANKRD42 reverse splicing biogenesis.** (**A**) qRT-PCR result illustrated that hucMSCs treatment reduced the expression level of circANKRD42 compared with TGF-β1/BLM treatment *in vivo* and *in vitro*. (**B**) Rescue experiment of cell proliferation showed that hucMSCs treatment reduced the TGF-β1-induced cell proliferation. circANKRD42 overexpression increased the cell proliferation and reversed the downward trend caused by hucMSCs treatment. (**C**) Rescue experiment of cell scratch assay monitored with an IncuCyte S3 instrument validated that hucMSCs treatment blocked the cell migration. circANKRD42 overexpression promoted the cell migration and reversed the downward trend caused by hucMSCs treatment. (**D**) Rescue experiment showed that hucMSCs treatment reduced the expression of S100A4, FAP1, α-SMA, vimentin, and collagen I and III. circANKRD42 overexpression increased the expression of S100A4, FAP1, α-SMA, vimentin, and collagen I and III and reversed the downward trend caused by hucMSCs treatment. (**E**) Immunofluorescence images exhibited that hnRNP L was located in the nucleus and its expression decreased in the hucMSCs treatment group compared with that in the TGF-β1 group. (**F**) Example of hnRNP L immunofluorescence images depicted that hnRNP L expression was increased in patients with IPF compared with that in normal individuals. (**G**) Western blot analysis identified that hucMSCs treatment inhibited hnRNP L expression compared with that in the TGF-β1/BLM group *in vivo* and *in vitro*. (**H**) RIP uncovered that hnRNP L bound to pre-mRNA (ANKRD42) and hucMSCs treatment reduced the binding amount of hnRNP L with pre-mRNA (ANKRD42). (**I**) qRT-PCR was performed to prove that the inhibitory effect of hucMSCs on circANKRD42 depending on hnRNP L. (**J**) MiR-136-5p expression markedly decreased in the TGF-β1/BLM group and increased in the hucMSCs treatment group *in vivo* and *in vitro*. (**K**) RAP experiment showed that miR-136-5p was enriched in circANKRD42; this enrichment was intensified by TGF-β1 and decreased by hucMSCs treatment. Lac Z was the control. BP indicates blank plasmid, and RP indicates the recombinant plasmid of overexpressed circANKRD42. Each bar represents the mean ± SD; *n* = 6; ^*^p < 0.05.

The upstream signal pathway of YAP1 was further explored. As a regulator of mechanical cue-driven YAP1 signal, circANKRD42 can target YAP1 by sponging miR-136-5p [5]. Hence, the regulatory mechanism of hucMSCs treatment on circANKRD42–miR-136-5p–YAP1 signal axis was explored. qRT-PCR results illustrated that circANKRD42 expression was decreased by hucMSCs treatment *in vivo* and *in vitro* (Figure 5A). Overexpressed circANKRD42 plasmid was synthesized and transfected into MRC-5 cells. The rescue experiment of cell proliferation analysis revealed that hucMSCs treatment reduced the TGF-β1-treated cell proliferation. circANKRD42 overexpression increased the cell proliferation and reversed the downward trend caused by hucMSCs treatment (Figure 5B). The rescue experiment of cell scratch assay monitored with an IncuCyte S3 instrument validated that hucMSCs treatment blocked the migration. circANKRD42 overexpression promoted the migration and reversed the downward trend caused by hucMSCs treatment (Figure 5C). The rescue experiment of Western blot indicated that circANKRD42 overexpression reversed the downward expression of S100A4, FAP1, α-SMA, vimentin, and collagen I and III caused by hucMSCs treatment (Figure 5D). These series of rescue experiments indicated that hucMSCs treatment alleviated pulmonary fibrosis by downregulating circANKRD42 *in vitro*. Further exploration was conducted on the circANKRD42 regulatory mechanism of hucMSCs treatment. Heterogeneous nuclear ribonucleoprotein L (hnRNP L) is an alternative RNA splicing factor involved in RNA splicing biogenesis [21]. Immunofluorescence images depicted that hnRNP L was located in the nucleus with or without hucMSCs/TGF-β1 treatment. hucMSCs treatment inhibited hnRNP L expression (Figure 5E). Sample immunofluorescence images confirmed that hnRNP L expression increased in patients with idiopathic pulmonary fibrosis (IPF) compared with that in normal individuals (Figure 5F), proving that the *in vivo* and *in vitro* results were credible. Western blot analysis also verified that hucMSCs treatment inhibited hnRNP L expression (Figure 5G). RNA-binding protein immunoprecipitation experiment was performed to uncover the effect of hucMSCs treatment on the binding of circANKRD42 with hnRNP L. The results showed that hnRNP L bound to pre-mRNA (ANKRD42) in pulmonary fibrosis and hucMSCs treatment reduced the binding amount of hnRNP L with pre-mRNA (ANKRD42) (Figure 5H). The rescue experiment of qRT-PCR was performed to prove that the inhibitory effect of hucMSCs on the circANKRD42 depending on hnRNP L, the results showed that the high expression level of circANKRD42 in TGF-β1 group decreased after hucMSCs treatment, and the expression level of circANKRD42 increased again after overexpression of hnRNP L. (Figure 5I). The above data suggested that hucMSCs treatment prevented circANKRD42 reverse splicing biogenesis by inhibiting hnRNP L expression.

qRT-PCR was performed to prove that the regulatory mechanism of hucMSCs treatment is via the upstream circANKRD42-miR-136-5p of YAP1. The results verified that hucMSCs treatment enhanced miR-136-5p expression compared with TGF-β1/BLM (Figure 5J). RNA antisense purification analysis showed that miR-136-5p was enriched in circANKRD42. This enrichment was further intensified by TGF-β1 but was decreased by hucMSCs treatment (Figure 5K), indicating that hucMSCs treatment depended on circANKRD42-miR-136-5p signal pathway *in vitro*.

**Figure 6:**
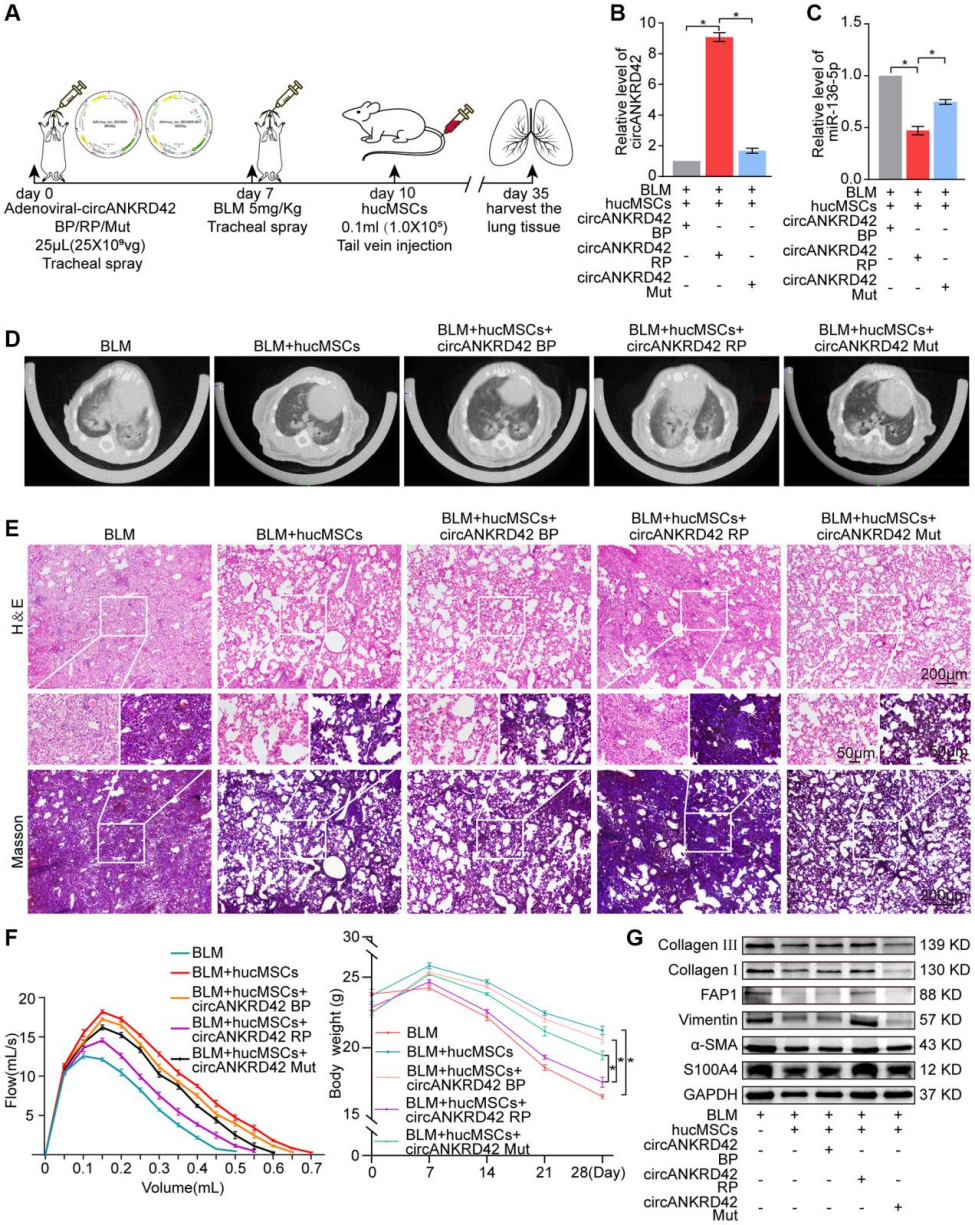
**Rescue experiments proved that hucMSCs treatment alleviated pulmonary fibrosis by regulating the upstream circANKRD42-miR-136-5p of YAP1 in mice.** (**A**) Schematic of the administration of overexpressed circANKRD42, circANKRD42 mutation, BLM, or hucMSCs into mice. (**B**) qRT-PCR revealed that circANKRD42 was substantially upregulated in overexpressed circANKRD42 group compared with other groups and reversed the inhibitory effect of hucMSCs treatment. The effect of circANKRD42 mutation was weaker than that of overexpressed circANKRD42. (**C**) circANKRD42 overexpression reversed the upward trend of miR-136-5p caused by hucMSCs treatment, and the effect of circANKRD42 mutation on miR-136-5p was weaker than that of overexpressed circANKRD42. (**D**) MicroCT imaging system for small animal showed that circANKRD42 overexpression reversed the therapeutic effect of hucMSCs and aggravated the degree of fibrosis. The effect of circANKRD42 mutation on fibrosis was weaker than that of overexpressed circANKRD42. (**E**) H&E and Masson staining exhibited that BLM caused excessive collagen deposition, bulk fibroblast paraplasm displacing alveolar space in lung tissues, and near disappearance of alveoli pulmonis. Fibrosis was improved in the BLM+hucMSCs group. Overexpressed circANKRD42 reversed the therapeutic effect of hucMSCs, but the effect of circANKRD42 mutation was weaker than that of overexpressed circANKRD42. (**F**) BLM deteriorated the lung function and body weight. Overexpressed circANKRD42 reversed the ameliorating effect of hucMSCs on the lung function and body weight. The effect of circANKRD42 mutation on the lung function and body weight was weaker than that of overexpressed circANKRD42. (**G**) Western blot identified that BLM promoted the expression levels of fibrotic and differentiation proteins. Overexpressed circANKRD42 reversed the effect of hucMSCs on these proteins. The effect of circANKRD42 mutation was weaker than that of overexpressed circANKRD42. Each bar represents the mean ± SD; *n* = 6; ^*^p < 0.05.

ATGGAG was mutated to CTTGCG (the binding site of miR-136-5p) in the full-length sequence of circANKRD42 to further confirm that hucMSCs treatment alleviates pulmonary fibrosis by regulating circANKRD42-miR-136-5p. circANKRD42 mutant and overexpressed circANKRD42 were packaged into the adenovirus vectors and sprayed into the lungs of mice (Figure 6A). qRT-PCR showed that circANKRD42 expression was substantially increased in the overexpressed circANKRD42 group compared with that in the other groups. In addition, the inhibitory effect of hucMSCs treatment on circANKRD42 was reversed. The effect of circANKRD42 mutation was weakened compared with that of overexpressed circANKRD42 (Figure 6B). MiR-136-5p expression was markedly decreased in the BLM group and increased in the hucMSCs treatment group. circANKRD42 overexpression reversed the upward trend of miR-136-5p caused by hucMSCs treatment. The effect of circANKRD42 mutation on miR-136-5p was weaker than that of overexpressed circANKRD42 (Figure 6C). These results indicated that the effect of hucMSCs treatment on circANKRD42 depended on miR-136-5p *in vivo.*

Rescue experiments of MicroCT imaging observation, H&E and Masson staining, lung function and body weight test, and Western blot were performed to further prove that hucMSCs treatment alleviated pulmonary fibrosis by regulating circANKRD42-miR-136-5p *in vivo*. MicroCT image clarified that BLM aggravated the degree of fibrosis and caused the thickening of the alveolar wall. hucMSCs exhibited a therapeutic effect against fibrosis, but this action was reversed by the overexpressed circANKRD42. The effect of circANKRD42 mutation on fibrosis was weaker than that of overexpressed circANKRD42 (Figure 6D). H&E and Masson staining exhibited that BLM caused excessive collagen deposition, bulk fibroblast paraplasm displacing alveolar space in lung tissues, and near disappearance of alveoli pulmonis. Fibrosis was improved in the BLM + hucMSCs group, but overexpressed circANKRD42 reversed the therapeutic effect of hucMSCs. The effect of circANKRD42 mutation was weaker than that of overexpressed circANKRD42 (Figure 6E). Similarly, BLM deteriorated the lung function and body weight. Overexpressed circANKRD42 reversed the ameliorating effect of hucMSCs on the lung function and body weight. The effect of circANKRD42 mutation on the lung function and body weight was weaker than that of overexpressed circANKRD42 (Figure 6F). The rescue experiment of Western blot also showed that BLM promoted the expression levels of fibrotic and differentiation proteins, and overexpressed circANKRD42 reversed the effect of hucMSCs on these proteins. The effect of circANKRD42 mutation on these proteins was weaker than that of overexpressed circANKRD42 (Figure 6G).

### hucMSCs treatment reducing mechanical stiffness by regulating circANKRD42–miR-136-5p–YAP1 signal pathway

**Figure 7:**
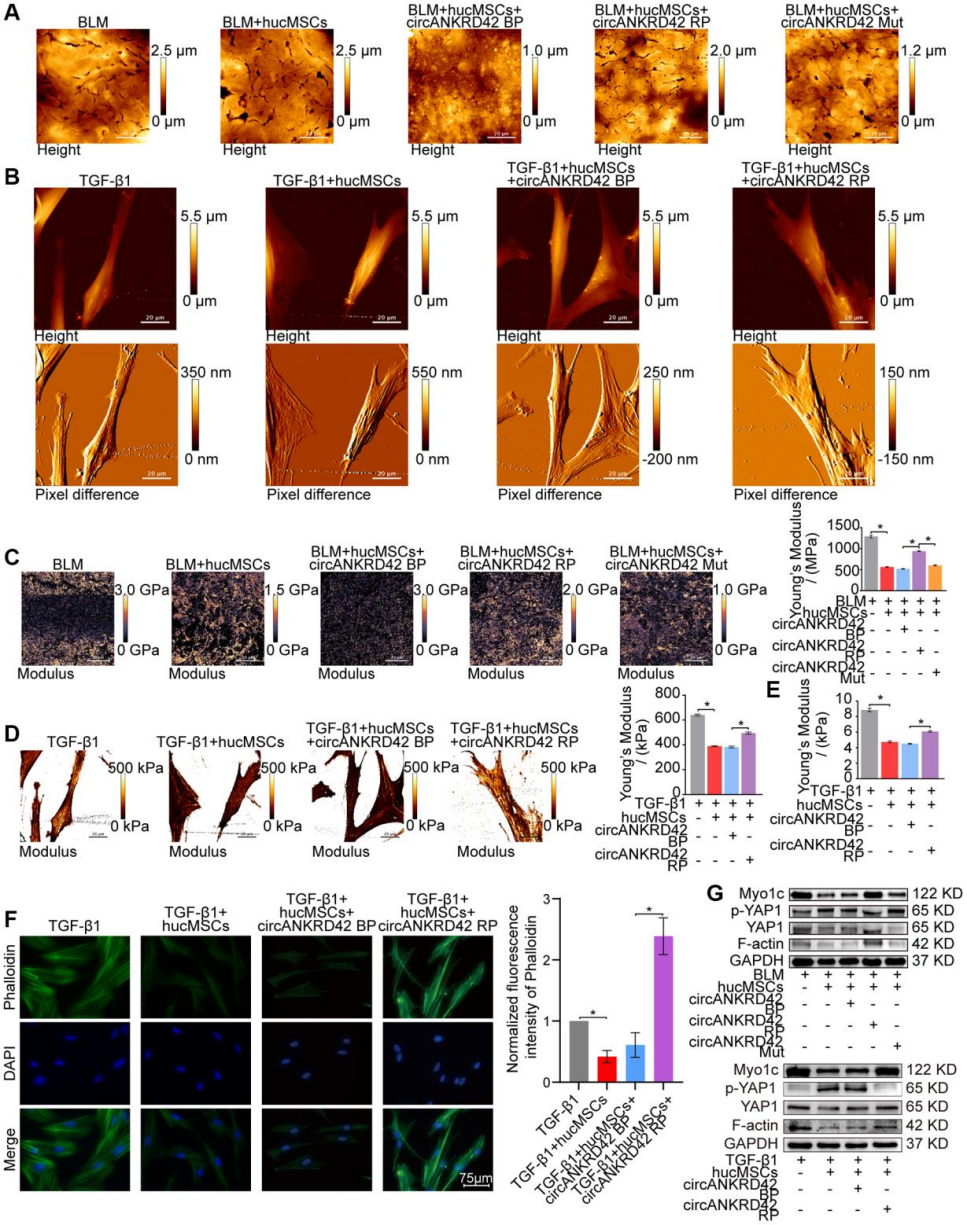
**Rescue experiments proved that hucMSCs treatment reduced mechanical stiffness by regulating circANKRD42–miR-136-5p–YAP1 signal pathway *in vivo* and *in vitro*.** (**A**) AFM images depicted that BLM caused the roughness of the lung surface and the thickening of the lung tissues. hucMSCs treatment improved the lung tissue morphology, but this therapeutic effect was reversed by overexpressed circANKRD42. The effect of circANKRD42 mutation was weaker than that of overexpressed circANKRD42. (**B**) In the TGF-β1 group, the cells became slender and flat. Stress fibers arranged in parallel along the main axis of cells were observed in the group. In the hucMSCs-treated group, the cell shape significantly retracted and the corresponding fibers were reduced. Overexpressed circANKRD42 reversed the effect of hucMSCs treatment on cells. (**C**) Young’s modulus measurement showed that hucMSCs treatment decreased the force compared with that in the BLM group, but overexpressed circANKRD42 reversed the effect of hucMSCs treatment on the force. The effect of circANKRD42 mutation on the force was weaker than that of overexpressed circANKRD42. The measured average Young’s modulus was 1291.667 ± 82.735 MPa in the BLM group, 563.833 ± 31.822 MPa in the BLM+hucMSCs group, 520 ± 20.460 MPa in the BLM+hucMSCs+circANKRD42 BP group, 942.05 ± 28.203 MPa in the BLM+hucMSCs+circANKRD42 RP group, and 604.233 ± 29.259 MPa in BLM+hucMSCs+circANKRD42 Mut. (**D**) Young’s modulus measurement revealed that hucMSCs treatment decreased the force compared with that in the TGF-β1 group, but overexpressed circANKRD42 reversed the effect of hucMSCs treatment on the force. The measured average Young's modulus was 641.167 KPa in the TGF-β1 group, 389.467 KPa in the TGF-β1+hucMSCs group, 382.767 in the TGF-β1+hucMSCs+circANKRD42 BP group, and 495.433 KPa in TGF-β1+hucMSCs+circANKRD42 RP. (**E**) Young’s modulus measurement showed that hucMSCs treatment decreased the reaction forces of cells compared with that in the TGF-β1 group, but overexpressed circANKRD42 reversed the effect of hucMSCs treatment on the force. The measured average Young's modulus was 8.858 ± 0.526 KPa in the TGF-β1 group, 4.756 ± 0.306 KPa in the TGF-β1+hucMSCs group, 4.504 ± 0.160 KPa in the TGF-β1+hucMSCs+circANKRD42 BP group, and 6.092 ± 0.240 KPa in TGF-β1+hucMSCs+circANKRD42 RP. (**F**) Cytoskeleton staining with FITC-phalloidin depicted that cytoskeleton tension was weakened by hucMSCs treatment but enhanced by circANKRD42 overexpression. (**G**) hucMSCs treatment inhibited the expression levels of YAP1, Myo1c, and F-actin and increased p-YAP1 expression *in vivo* and *in vitro*. circANKRD42 overexpression increased the expression of YAP1, Myo1c, and F-actin and decreased p-YAP1 expression, thus reversing the effect of hucMSCs treatment *in vivo* and *in vitro*. The effect of circANKRD42 mutation was weaker than that of overexpressed circANKRD42. Each bar represents the mean ± SD; *n* = 6; ^*^p < 0.05.

Rescue experiments were performed to prove that hucMSCs treatment reduces mechanical stiffness by regulating circANKRD42–miR-136-5p–YAP1 signal pathway *in vivo* and *in vitro*. AFM images depicted that BLM caused the roughness of the lung surface and the thickening of the lung tissues. hucMSCs treatment improved the lung tissue morphology, but this therapeutic effect was reversed by overexpressed circANKRD42. The effect of circANKRD42 mutation on lung tissues was weaker than that of overexpressed circANKRD42 (Figure 7A). Stress fibers arranged in parallel along the main axis of cells were found in the TGF-β1 group. After hucMSCs intervention, these fibers were reduced. Overexpressed circANKRD42 reversed the effect of hucMSCs treatment on fibers (Figure 7B). In terms of Young’s modulus, hucMSCs treatment decreased the force compared with that in the BLM/TGF-β1 group, but overexpressed circANKRD42 reversed the effect of hucMSCs treatment on the force. The effect of circANKRD42 mutation on force was weaker than that of overexpressed circANKRD42 (Figure 7C, 7D). Tests using colloid probe with ball stuck pressed cells were conducted to determine the reaction forces of cells based on Young’s modulus. The result showed that hucMSCs treatment decreased the reaction forces of cells compared with those in the TGF-β1 group, but overexpressed circANKRD42 reversed the effect of hucMSCs treatment on the force measurement (Figure 7E). Cytoskeleton staining showed that hucMSCs treatment reduced the cytoskeleton tension compared with that in the TGF-β1 group, and overexpressed circANKRD42 reversed the effect of hucMSCs treatment on the cytoskeleton tension (Figure 7F). Mechanical stiffness-related proteins such as YAP1/p-YAP1, Myo1c, and F-actin were measured by Western blot *in vivo* and *in vitro*. The same results were obtained from the cell model and animal model. hucMSCs treatment inhibited the expression levels of YAP1, Myo1c, and F-actin and promoted p-YAP1 expression. circANKRD42 overexpression increased the expression of YAP1, Myo1c, and F-actin and decreased p-YAP1 expression, thus reversing the effect of hucMSCs treatment. The effect of circANKRD42 mutation on these proteins was weaker than that of overexpressed circANKRD42 (Figure 7G).

## DISCUSSION

hucMSCs therapy provides a promising approach against intractable diseases in clinical trials due to the immunomodulatory properties and regeneration ability of hucMSCs. For example, hucMSCs infusion for patients with COVID-19 potentially improves clinical symptoms, such as short hospital stay, less time required for symptoms remission, and few adverse events [22–24]. hucMSCs treatment improved the immune reconstitution in 72 immune nonresponder patients with chronic HIV-1 infection [25]. A retrospective analysis on 18 patients showed that robot-assisted core decompression combined with hucMSCs therapy is a feasible and relatively safe method for the treatment of femoral head necrosis [26]. Yao et al. first systematically demonstrated the clinical safety and efficacy of hucMSCs in treating neuromyelitis optica spectrum disorder and determined the optimal dose of hucMSCs for patients with this illness [27]. He et al. showed that the safe dosage of repeated intravenous infusion of cynomolgus monkey umbilical cord mesenchymal stem cells in cynomolgus monkeys is 1.0 × 10^7^/kg, which is 10 times of that in clinical human use [28]. In some follow-up study the data suggested that combined IV of hucMSCs transplantation is safe and feasible [29–31]. hucMSCs therapy has also been studied for its clinical therapeutic potential against other diseases, including refractory uveitis [32], chronic ischemic cardiomyopathy [33], relapse remitting multiple sclerosis [29], and chronic ischemic heart disease [34]. Unfortunately, the clinical trial for 18 patients with lupus nephritis was abandoned because hucMSCs therapy failed to exhibit a positive treatment effect [35]. To the authors’ knowledge, only one patient with IPF underwent hucMSCs treatment in clinical trials [36]. One of the reasons is the lack of basic study in pulmonary fibrosis field, hindering the development of clinical practice for hucMSCs treatment. HucMSCs treatment can inhibit systemic inflammatory responses in mice [37]. Shi et al. showed that histopathological examination of the spleens and thymi from rats in each group had no significant changes when a single injection of hucMSCs into rat ovaries [38]. Ding et al. showed that in a xenograft rejection assay, the hucMSCs survived in immunocompetent mice, whereas primary fibroblasts did not survive. This study confirmed the HLA-G-related immunosuppressive property of hucMSCs [39]. It was also found in our experimental results that hucMSCs could survive for more than 28 days in mice. Moreover, in the present study, hucMSCs exhibited targeting lung property. hucMSCs therapy alleviated pulmonary fibrosis by reducing circANKRD42-YAP1-mediated mechanical stiffness during fibroblast to myofibroblast differentiation.

Mechanical signals are fundamental regulators of cell behavior, but how mechanical cues are sensed and transduced at the molecular level to regulate gene expression has long remained enigmatic [40]. Fibroblasts define the architecture of lung and can differentiate into myofibroblast, the main effector cells of pulmonary fibrosis [41–43]. Using IPF patient-derived cell cultures, Jaffar et al. [44] measured the stiffness of individual lung fibroblasts via high-resolution force maps with AFM and found that the fibroblasts from patients with IPF were stiffer and had an augmented cytoskeletal response to TGF-β1 compared with the fibroblasts from donors without IPF. In the present work, AFM images showed that the differentiated fibroblasts had increased cell density and matrix protein deposition and enhanced cell/tissue stiffness and cytoskeletal tension. hucMSCs treatment decreased cell density and matrix protein deposition, thus reducing cell/tissue stiffness and cytoskeletal tension. Accompanying these visible stiffness changes, the mechanochemical activation effect was generated, that is, the extracellular and intracellular stiffness initiated mechanical cues that activated intracellular signaling through stiffness–cell interactions. Our data demonstrated that YAP1 orchestrated responses between mechanical and biochemical signals.

**Figure 8:**
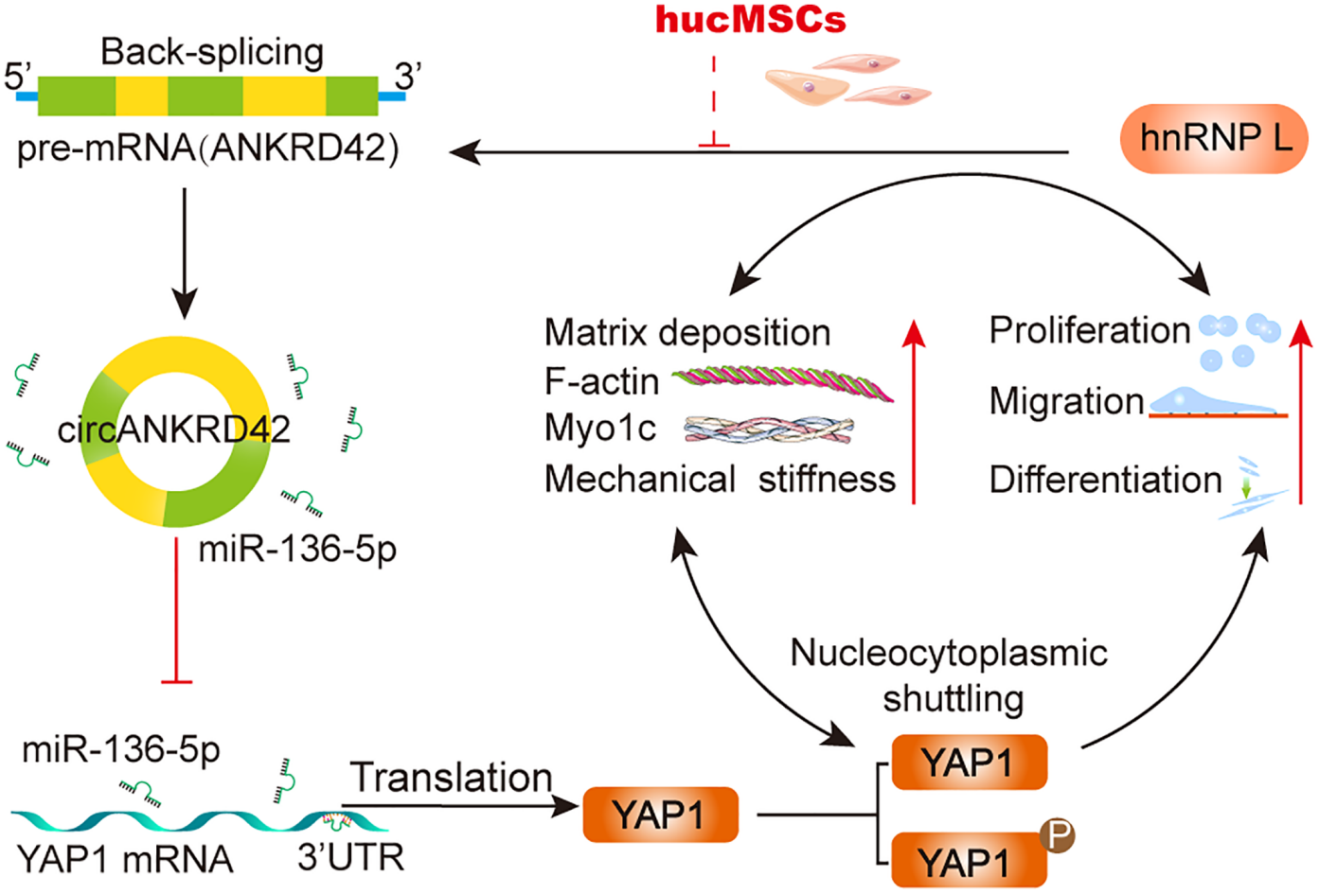
The mechanical stiffness mechanism of hucMSCs treatment in pulmonary fibrosis.

As a transcription coactivator, YAP1 is the sensor of mechanical and biochemical signal [45, 46]. circRNAs, such as circ1662, circ_0024093 and circRNA_104075, act as the upstream of YAP1 to control cell proliferation, differentiation, and migration by accelerating YAP1 nuclear localization [47–49]. The interaction between circRNA and YAP1 has the potential to serve as biomarker and therapeutic target in clinical applications [50]. However, whether the interaction can be a therapeutic target of stem cell therapy has never been explored. Our study showed that hucMSCs treatment alleviated pulmonary fibrosis by reducing circANKRD42-YAP1-mediated mechanical stiffness. Mechanistic dissection revealed that hucMSCs treatment repressed circANKRD42 reverse splicing biogenesis by inhibiting hnRNP L, which in turn promoted miR-136-5p to bind YAP1 and thus block YAP1 nuclear entry. The condition repressed the expression of related mechanical genes to block the force transmission and reduce the mechanical forces (Figure 8).

In conclusion, our results unveiled the mechanosensing mechanism mediated directly by circANKRD42-YAP1 axis in hucMSCs treatment, which has potential general applicability in pulmonary fibrosis treatment. We hope that this study will provide novel therapeutics to combat pulmonary fibrosis.

## MATERIALS AND METHODS

### hucMSCs isolation and culture

The study was approved by the Ethics Committee of Binzhou Medical University Hospital (NO. 2020-G031-01). The umbilical cords were donated after obtaining the consent of the pregnant women. hucMSCs were isolated from the human umbilical cord of full-term newborn and cultured in Dulbecco's modified Eagle's medium containing low glucose (DMEM-LG, Hyclone, USA) supplemented with 10% fetal bovine serum (FBS, Hyclone, USA), 100 U/mL penicillin G and 100 U/mL streptomycin sulfate and incubated at 37°C with 5% CO_2_.

### Adipogenic and osteogenic differentiation

The third generation of hucMSCs was seeded in 24-well plates. When the cells grow to 70-90% fused, the medium was replaced by human umbilical cord marrow mesenchymal stem cells adipogenic differentiation kit (Cas9X™, UCHX-D102) or human umbilical cord marrow mesenchymal stem cells osteogenic differentiation kit (Cas9X™, UCHX-D101). The cells were cultured in 37°C, 5% CO2 incubator. The medium was replaced per 3 days. After induction of 28 days or 14 days, oil red O or alizarin red staining were performed and observed under microscope.

### Flow cytometry analysis

Cultured hucMSCs were centrifuged at 1000 rpm for 5 min. After supernatant was discarded, 1×PBS (Sparkjade, CR0013) was added to resuspend precipitate. CD90 (Biolegend, B317126), CD73 (Biolegend, B322682), CD45 (Biolegend, B286628), CD44 (Biolegend, B285080), CD34 (Biolegend, B320957), and HLA-DR (Biolegend, B275368) antibodies were added, respectively. After incubation at 4°C for 30 min, 1 mL 1×PBS was added to wash away the unbound antibodies. Flow cytometry was used to detect expression of these antibodies.

### Animal model

C57BL/6 male mice aged 8 weeks, with an average body weight of 22 ± 3 g, were purchased from Jinan Pengyue Experimental Animal Breeding Company (Jinan, China). According to the experimental requirements, the mice were randomly divided into different groups (10 mice in each group) using a simple random allocation method: sham, bleomycin (BLM), sham+hucMSCs, BLM+hucMSCs treatment, and BLM+hucMSCs+circANKRD42 blank plasmid (BP)/ recombinant plasmid (RP)/mutation (Mut) group. The BLM group was administered 5 mg/kg BLM which was sprayed into the lungs using a Penn-Century MicroSprayer (Penn-Century Inc., Wyndmoor, PA, USA), while the sham group was sprayed with the same amount of saline. The BLM+hucMSCs+circANKRD42 BP/RP/Mut group mice were administered 25 × 10^9^ vg adenoviral-circANKRD42 BP/RP/Mut which was also sprayed into the lungs using a Penn-Century MicroSprayer. About 0.1 mL hucMSCs (1.0 × 10^5^) were injected into mice through the tail vein in the hucMSCs treatment group after three days of BLM spraying. On the 28th day of modeling, the lung changes of all mice were evaluated by a MicroCT imaging system for small animals (PerkinElmer, USA). Mice were anesthetized by intraperitoneal injection of 2.5% Avertin (0.25 g/kg). Then, lung samples were collected, processed and stored for the following experiments. Experiments on the animals were approved by the Animal Experiments Ethics Committee of Binzhou Medical University Hospital (No. 20200128-19).

### hucMSCs-targeted lung experiment

hucMSCs-targeted lung experiment was performed using the cell plasma membrane staining kit with DiI (Beyotime, Shanghai, China) according to the manufacturer’s protocol. In brief, 10 μM DiI dye was added into the hucMSCs at 37°C for 20 min. Then the hucMSCs were washed by 1×PBS and centrifuged at 1000 rpm for 5 min. The supernatant was discarded. The hucMSCs were diluted to 1.0 × 10^6^/mL in serum-free medium and injected 0.1 mL cell suspension into mice through tail vein. The lung tissues of mice were harvested at 3, 7, 14, 21 and 28 days after injection. The frozen slices were obtained by using freezing microtome (Leica CM1950, Germany) and photographed under fluorescence microscope.

### Cell culture and treatment

Human fetal lung fibroblast MRC-5 cell line was obtained from American Type Culture Collection (CCL-171™) and cultured in minimum essential medium (MEM, Gibco, 11090081) supplemented with 10% FBS (Gibco, 10270106), 1% GlutaMax™ (Gibco, 35050061), 1% NEAA (Gibco, 11140050), 1% Sodium Pyruvate (Gibco, 11360070), and 100× penicillin/streptomycin solution (SparkJade, CM0004) at 37°C, 5% CO^2^ incubator. Cells were divided into different groups according to the experimental requirements: normal, TGF-β1, normal+hucMSCs, TGF-β1+hucMSCs, TGF-β1+hucMSCs+circANKRD42 BP/RP. Cells were treated with or without 5 ng/mL TGF-β1 (Gibco, PHG9202) and/or co-cultured with hucMSCs in tissue culture plate insert (JetBiofil, TCS001006) for 72 h in the normal, TGF-β1, normal+hucMSCs and TGF-β1+hucMSCs group. The overexpressed circANKRD42 group cells were transfected with circANKRD42 BP plasmid (OBiO, Shanghai, China) for 24 h.

### Western blot

Tissues or cells were collected and lysed in radioimmunoprecipitation assay (RIPA, Solarbio, R0020) buffer and phenylmethylsulfonyl fluoride (PMSF, Solarbio, P0100) (RIPA buffer: PMSF = 100:1). The protein concentration was measured by bicinchoninic acid protein assay kit (Coolaber, SK1070). After separated in sodium dodecyl sulfate polyacrylamide gel electrophoresis, the protein was transferred to polyvinylidenefluoride membranes. The protein was sealed for 2 h with 5% skim milk and incubated overnight at 4°C with antibodies: anti-collagen I (Affinity, AF7001), anti-collagen III (Affinity, AF0136), anti-vimentin (Affinity, AF7013), anti-α-SMA (Affinity, AF1032), anti-FAP1 (Cell Signaling Technology, 66562S), anti-S100A4 (Cell Signaling Technology, 13018S), anti-Myo1c (Abcam, ab194828), anti-YAP1 (Cell Signaling Technology, 14074S), anti-phospho-YAP1 (Cell Signaling Technology, 53749S), anti-F-actin (Abcam, ab130935), anti-GAPDH (Affinity, AF7021). 1×Tris buffered saline Tween was used to wash the membranes for three times. Then membranes were incubated for 1 h with secondary antibodies at room temperature. The expression of proteins was detected by enhanced chemiluminescence reagent kit (SparkJade, ED0015-B).

### qRT-PCR

Total RNA was isolated from cells or tissues using AG RNAex Pro reagent (Accurate Biology, AG21102). Complementary DNA synthesis was performed using Evo M-MLV RT Premix reagent (Accurate Biology, AG11706) following the manufacturer`s instructions. The qRT-PCR was performed using a 2×SYBR Green Pro Taq HS Premix (Accurate Biology, A3A2291) on the Rotor Gene3000 real-time PCR system. The reaction system was 20 μL and conditions were as follows: pre-denaturation at 95°C for 10 minutes and PCR amplification for 45 cycles at 95°C for 5 seconds, 60°C for 30 seconds and 72°C for 30 seconds. The primers for miR-136-5p were obtained from Guangzhou RiboBio Co. Ltd. (Guangdong, China). The forward and reverse primers of circANKRD42 are as follows: circANKRD42-F 5'-GGAGAGACCCAGTGATGTGG-3' and circANKRD42-R 5'-TCATCCTGGGCTGTCAGATTTGCTC-3'.

### Cellular proliferation and migration analysis

1 × 10^6^/mL cells were seeded in a 6 well cell culture plate (Corning, REF3516) for proliferation analysis and 5 × 10^4^/mL cells were seeded in 96 well cell culture plate (Corning, REF3599) for migration analysis, respectively. The WoundMaker was used to create precise and reproducible wounds in all wells in migration analysis. Then cell samples were placed in IncuCyte S3 live-cell analysis system (Essen BioScience, USA) for real-time dynamic observation, which automatically records a real-time cellular proliferation or migration. Schedule repeat scanning was taken on IncuCyte S3 software and analysed with IncuCyte 2021A software.

### Cytoskeleton staining

The cell samples were seeded on circular slides, placed in 24-well plates and treated in different group, then fixed with cell fixative including 4% paraformaldehyde (Meilunbio, MA0192) for 30 minutes. After being washed with 1×PBS three times, the cell samples were incubated with 0.1% TritonX-100 (Sinopharm, 30188928) for 8 minutes. Then 200 μL of 1% AbFluor™ 488-Phalloidin (Abbkine, BMD00082) was added for 30 minutes. Then samples were washed with 1×PBS and added DAPI (Sigma, D9542-1MG) for 7 minutes. Finally, cytoskeleton was observed by using automatic living cell fluorescence microscopic imaging system (Invitrogen, EVOS M5000).

### AFM imaging and Young's modulus determination

MRC-5 cells (1.0 × 10^5^) were cultured at WillCo-dish^®^ glass bottom dishes (Willco Wells BV, GWST-5040) and subjected to an atomic force microscope (JPK NanoWizard 4, Bruker Nano GmbH) for imaging in QI mode. The topographical images were acquired in PBS by using a PFQNM-LC-CAL probe (Bruker Nano GmbH) with an end radius of 75 nm and a force constant around 0.09 N/m. After an entire cell was imaged by AFM in PBS, the colloid probe (MLCT-O-A probe, Bruker Nano GmbH) with a 20-μm diameter silica sphere was localized on an intact single cell, and then the mechanical stiffness (i.e., Young’s modulus) was measured by Hertz model via AFM indentation test. The Young’s modulus distributions of the lung tissue of mouse were characterized on an atomic force microscope (JPK NanoWizard 4, Bruker Nano Inc.). Frozen sections of mouse lung tissue were placed on slides. The topographies were measured by QI mode, which collected force curve matrix for further calculation of Young’s modulus distribution. A ScanAsyst-Fluid probe with a force constant around 0.7 N/m and a tip radius around 20 nm was applied in the characterization. Force curves in matrix were fitted with Hertz model to calculate the corresponding Young’s modulus by using commercial software provided from Nano Wizard 4.

### RNA antisense purification (RAP)

The RAP was performed under the guidance of BersinBioTM RNA antisense purification kit (BersinBio, Bes5103-3). The cells were collected, cross-linked with 1% formaldehyde PBS and 1.375 M Glycine, lysed with lysis buffer, protease inhibitor and RNase inhibitor. DNase salt stock, DNase, EDTA, EGTA and DTT were added to remove DNA. The supernatant was collected after centrifugation at 4°C 16,000 g for 10 min. At the same time, the probe and beads were pretreated with 10 mM Tris-HCl and 1×Hybridization buffer. Then, the supernatant was incubated at 65°C, 10 min to denaturation. Probe was added to the denatured samples at 37 °C, 30 min, 50 °C, 5 min, and hybridized for 120 min. Samples were added with RNA elution buffer, 5×Proteinase K buffer, proteinase K, phenol-chloroform-isoamyl alcohol mixture (25:24:1), NaCl, Glycogen, absolute ethanol and RNase-free water for RNA elution and purification. Finally, RNA was reversed transcription and PCR. The qRT-PCR conditions were as follows: holding temperature at 95°C for 10 min. 45 cycles of PCR amplification at 95°C for 5 s, 60°C for 30 s and 72°C for 30 s. The probes of Lac Z (negative control) and hsa_circANKRD42 were: GCCTGATGCGGTATTTTCTCCTTACGCATCTGTGCGGTATTTCACACCGCATATGGTGCA and AGCTCCATGCCAGAGCAGCCAATGAAGACACTCTTTGCCACATCACTGGGTCTCTCCCCT, respectively.

### Statistical analysis

Statistical analyses were performed using SPSS version 19.0 software. Data were presented as the mean ± SD of at least three independent experiments. Unpaired Student’s *t* test was used for experiments comparing two groups, whereas one-way ANOVA with Student-Newman-Keuls post hoc test was applied for experiments comparing three or more groups. Statistical significance was considered at *p* < 0.05.

### Availability of data and materials

All data associated with this study are present in the paper.
